# Interactions between Social/ behavioral factors and *ADRB2* genotypes may be associated with health at advanced ages in China

**DOI:** 10.1186/1471-2318-13-91

**Published:** 2013-09-09

**Authors:** Yi Zeng, Lingguo Cheng, Ling Zhao, Qihua Tan, Qiushi Feng, Huashuai Chen, Ke Shen, Jianxin Li, Fengyu Zhang, Huiqing Cao, Simon G Gregory, Ze Yang, Jun Gu, Wei Tao, Xiao-Li Tian, Elizabeth R Hauser

**Affiliations:** 1 Center for the Study of Aging and Human Development, Geriatrics Division of School of Medicine, Duke University, Box 3003 Durham, NC 27710, USA; 2 Center for Healthy Aging and Development Studies, Duke University and National School of Development, Peking University, Beijing, China; 3 School of Business, Nanjing University, Nanjing, China; 4 Department of Human Population Genetics, Institute of Molecular Medicine, Peking University, Beijing, China; 5 Institute of Public Health, University of Southern Denmark, Odense, Denmark; 6 Department of Sociology, National University of Singapore, Singapore, Singapore; 7 Business School, Xiang Tan University, Xiangtan, China; 8 Institute of Population Research, Fudan University, Shanghai, China; 9 Department of Sociology, Peking University, Beijing, China; 10 Gene, Cognition and Psychosis Program, National Institute of Mental Health and Lieber Institute for Brain Development, Baltimore, MD, USA; 11 Center for Human Genetics, Medical School, Duke University, Durham, NC, USA; 12 National Institute of Geriatrics, Beijing Hospital, Ministry of Health of China, Beijing, China; 13 School of Life Sciences, Peking University, Beijing, China

**Keywords:** Health aging, Oldest-old, Social/behavioral factors, *ADRB2* genotypes, GxE Interactions, Cognitive function, Self-reported health, Regular exercise, Social-leisure activities, Negative emotion

## Abstract

**Background:**

Existing literature indicates that ADRB2 gene is associated with health and longevity, but none of previous studies investigated associations of carrying the ADRB2 minor alleles and interactions between ADRB2 genotypes and social/behavioral factors(GxE) with health outcomes at advanced ages. This study intends to fill in this research gap.

**Method:**

We conducted an exploratory analysis, using longitudinal survey phenotype/genotype data from 877 oldest-old aged 90+. To estimate association of GxE interactions with health outcome, adjusted for the potential correlation between genotypes and social/behavioral factors and various other potentially confounding factors, we develop and test an innovative three-step procedure which combines logistic regression and structural equation methods.

**Results:**

Interaction between regular exercise and carrying *rs1042718* minor allele is significantly and positively associated with good cognitive function; interaction between regular exercise and carrying *rs1042718* or *rs1042719* minor allele is significantly and positively associated with self-reported good health; and interaction between social-leisure activities and carrying *rs1042719* minor allele is significantly and positively associated with self-reported good health. Carrying *rs1042718* or *rs1042719* minor alleles is significantly and negatively associated with negative emotion, but the *ADRB2* SNPs are not significantly associated with cognitive function and self-reported health. Our structural equation analysis found that, adjusted for the confounding effects of correlation of the *ADRB2* SNPs with negative emotion, interaction between negative emotion and carrying *rs1042718* or *rs1042719* minor allele is significantly and negatively associated with cognitive function. The positive association of regular exercise and social-leisure activities with cognitive function and self-reported health, and negative association of negative emotion with cognitive function, were much stronger among carriers of *rs1042718* or *rs1042719* alleles, compared to the non-carriers.

**Conclusions:**

The results indicate significant positive associations of interactions between social/behavioral factors and the ADRB2 genotypes with health outcomes of cognitive function and self-reported health, and negative associations of carrying *rs1042718* or *rs1042719* minor alleles with negative emotion, at advanced ages in China. Our findings are exploratory rather than causal conclusions. This study implies that near-future health promotion programs considering individuals’ genetic profiles, with appropriate protection of privacy/confidentiality, would yield increased benefits and reduced costs to the programs and their participants.

## Background

The β-adrenergic system, which includes the β2-Adrenergic Receptor gene (ADRB2), is critical to the regulation of vascular tone [1], cell growth and apoptosis [2], lipid metabolism [3], and immunoresponse [4]. It was recently demonstrated in mice that the β-adrenergic system is associated with lifespan and resistance to stress [5]. A recent study of the Framingham Heart Study Offspring cohort (mean age 36.4 for men and 36.0 for women) found that the *ADRB2* gene appears to be associated with a broad range of aging-associated phenotypes, including cancers at different sites, myocardial infarction, intermittent claudication, and longevity [6]. They concluded that, in an evolutionary context, the *ADRB2* gene may play an important systemic role in healthy aging that warrants exploration in other populations. The authors stated, however, that a major limitation of their study was the very small sample of oldest-old individuals, preventing generalization of their conclusion to advanced ages [7].

Based on genotype data from 963 long-lived Han Chinese aged 90+ at baseline, most of whom survived to age 100+, and 1,028 middle-age controls, our group’s recent population association study identified two synonymous single-nucleotide polymorphisms (SNPs) of rs1042718 (C/A) and rs1042719 (G/C) that are significantly associated with longevity, namely, survivorship from middle-age to advanced ages (*P* = 0.001–0.0001, adjusted for gender) [8]. However, our prior study did not investigate associations of carrying the minor allele of *rs1042718* or *rs1042719* to specific health outcomes nor the association of interactions between genotypes and social/behavioral factors with health outcomes at advanced ages; we intend to address these research questions in this article. We first present a brief review of the relevant literature that sets up theoretically meaningful hypotheses.

Previous studies have shown that the *ADRB2* gene is associated with both longevity and specific psychological health outcomes; carriers are less likely to have panic disorder [9], hostility [10], psychomotor agitation [11], tension-anxiety [12], and depression [13]. The alleles of the *ADRB2* gene have recently been associated with autism in child twins [14] as well as in the Autism Genetic Resource Exchange (AGRE) cohort [15]. The β2-adrenergic receptor has also been found to be associated with Alzheimer’s disease [16,17].

Adrenergic receptor function or regulation is substantially associated with various social/behavioural stressors, such as physical or emotional stress [18,19], and hence interacts with these factors to produce a phenotype vulnerable to pathological states [19]. Several studies have investigated associations of the *ADRB2* gene and its interactions with the genotypes and social/behavioural exposures with survival among patients who suffered from specific diseases. Although we have learned a lot from these existing studies, they are limited by small sample sizes with a range from 22 to 210 individuals [7,18,20-26].

It is clear that the existing literature indicates associations of the ADRB2 gene with mental health, and that interactions between the ADRB2 gene and social/behavioral stressors (or stress releasers) may also be associated with mental health. Based on the existing literature and the genotype and phenotype data available to us, we intend to address the following exploratory research questions:

(1) Are carrying the minor alleles of *ADRB2* SNPs (rs1042718 or rs1042719) and its interactions with regular exercise or social/leisure activities, which are related to stress releasing, significantly associated with health outcomes at advanced ages, measured by cognitive function and self-rated health?

(2) Are carrying the rs1042718 or rs1042719 minor allele significantly associated with negative emotion (related to stress)? Are interactions between negative emotion and carrying the rs1042718 or rs1042719 minor allele significantly associated with cognitive function and self-rated health?

Note that some previous studies also indicated that, in general, genetic impacts on health and longevity are more profound at advanced ages [27], perhaps due to some unobserved and un-investigated heterogeneities including the effects of interactions between genetic and social/behavioural factors; but the other study found that the effects of APOE4 on mortality diminishes with age [28]. More importantly, the numbers of oldest-old have been increasing much more dramatically than any younger age groups in many countries while the oldest-old much more likely need health and daily living care. These facts imply that focusing on the oldest-old is a useful way to investigate the effects of genetics and their interactions with social/behavioural factors on healthy aging. However, almost all previous studies in this field focused on young and middle aged adults and few had large numbers of oldest-old subjects. We will address this limitation by examining genetic characteristics and gene-environment interactions among oldest-old adults in China.

## Methods

### Data sources

The phenotype and genotype data used in this study come from the 1998 baseline survey of the Chinese Longitudinal Healthy Longevity Survey (CLHLS). The CLHLS 1998 baseline survey was conducted among 8,959 oldest-old participants aged 80 and older in a randomly selected half of the counties and cities in 22 out of 31 provinces in China ^a^[29]. Extensive data were collected using an internationally standardized questionnaire adapted to the Chinese cultural and social context. Data included family structure, living arrangements and proximity to children, disability status, physical performance, self-rated health, life satisfaction, cognitive function, chronic diseases, medical care, social and leisure activities, diet, smoking, alcohol consumption, psychological characteristics, economic resources, and caregiving and family support [30].

The CLHLS 1998 baseline survey also collected blood dry-spot samples from willing participants. Genotypic data on the two SNPs of *rs1042718* and *rs1042719* were produced following internationally standardized technical procedures [8]. Details of the genotyping procedure; allelic association analysis; genotypic association analysis with dominant, recessive and additive models ^b^; and the linkage disequilibrium and haplotype association analysis were presented in a previous article [8], and will not be repeated in this article. Here we present a new statistical analysis using both the phenotype and genotype data for 877 Han Chinese (576 women and 301 men) aged 90+ ^c^. Although the CLHLS also interviewed respondents in various age groups, we only included participants aged 90+ in this study because the focus of this article is on advanced ages and the genotypic data are unavailable for participants in other age groups.

Careful evaluations, including reliability of coefficients and factor analysis, have shown that the data quality of the CLHLS surveys is reasonably good [29]. It has been well-established and re-confirmed by a wide variety of international comparative studies [31-33] that age reporting of the Han Chinese population, including the oldest-old, is relatively accurate due to the cultural tradition of memorizing date of birth to determine important life events such as dates of engagement, marriage, starting to build a residential house, etc. This Chinese cultural tradition was especially prevalent for those born more than 90 years ago.

All of the genotypic and phenotypic data used in this study are from participants who belong to the same ethnic group of Han Chinese in China. Note that, unlike the U.S. and other Western countries which received many immigrants from other parts of the world and involve relatively heterogeneous genetic compositions even within the same racial group, China received very few international immigrants in the past decades. The Han Chinese thus represent a genetically homogenous population, which is a comparative advantage to avoid serious population stratification problems when studying influences of genetics and gene-social/behavioral interactions on health.

The Research Ethics Committees of Duke University and Peking University granted approval for the Protection of Human Subjects for each wave of the Chinese Longitudinal Healthy Longevity Survey, including DNA sample collection. The survey respondents gave informed consent before participating.

### Measurements

#### Dependent variables: health outcomes

##### Cognitive function measured by Mini-Mental State Examination (MMSE)

The MMSE, a global assessment of cognitive function [34], was adapted to the Chinese cultural context and was carefully tested in the pilot survey [30]. The questionnaire includes 24 items regarding orientation, registration, attention, calculation, recall and language, with a total score ranging from 0 to 30 ^d^. Following the previously adopted practice in the literature, we use the MMSE cutoffs to define cognitive function as “Good” (24+) “Moderate (21-23),” and “Poor” (<21) ^e^[30,35,36].

##### Self-reported health

Self-reported health was measured with the internationally standard question of “How do you rate your health at present?” Five possible answers included: very good, good, so so, bad, very bad, and not able to answer. Self-reported health (SRH) is considered to be good if the interviewee responds “good” or “very good”; otherwise, it is considered to be poor. SRH has often been used as an indicator of underlying health status in population health research; dichotomized SRH has been frequently used in previous studies [37-39]. Studies have shown that SRH is associated with mortality [40] and can be a better predictor of diverse aspects of well-being than clinical factors among older people [41,42].

Because all of the dependent variables used in this study (MMSE and SRH) are subjective, we did not use responses by proxy. Information on the independent variables described below (except negative emotion) could be provided by a proxy, such as an interviewee’s close family member, if the interviewee could not respond. As all of valid cases of the 877 oldest-old participants analyzed in this study had self-reported MMSE, SRH and negative emotion (the cases who were not able to self-report these subjective measures were eliminated), the proxy uses for the other non-subjective explanatory variables in this sample are very rare.

#### Independent variables

##### Primary independent variable

For each of the two SNPs of rs1042718 and rs1042719, the presence or absence of the minor allele is indicated by the label “carrier” if a person carries 1 or 2 copies of the minor allele, or “non-carrier” if he or she does not carry the minor allele, based on the dominant model (see endnote b).

In addition to the primary independent variable, we chose the following demographics and socioeconomic variables as covariates.

*Demographic and socioeconomic variables*, including *age, gender, residence* (rural vs. urban), and *education* (less than one year schooling vs. ≥1 year of schooling ^f^).

*Family support and connections*, including marital status and proximity to children*. Marital status* refers to currently married vs. unmarried (including never-married, divorced or widowed). *Proximity to children* is measured dichotomously: participants who live with their children or have at least one child living nearby in the same village or on the same urban neighborhood district, versus those who have neither co-resident children nor children living nearby.

Behavioral and social participation factors, including “negative emotion” (related to stress) and “regular exercise and social/leisure activities” (related stress releasing).

*Negative emotion* is an unhealthy and psychologically related social/behavioral factor associated with health outcomes, as indicated in the literature which found that, for example, negative emotion is negatively related with cognitive function [43]. We constructed a measure of negative emotion based on responses to three questions asked in the CLHLS: (1) Do you often feel the older you get, the more useless you are? (45.3% of oldest-old answered yes); (2) Do you often feel fearful or anxious? (14.6% of oldest-old answered yes); and (3) Do you often feel lonely and isolated? (17.4% of oldest-old answered yes). Interviewees who answered “always” or “often” for these questions were regarded as “yes”; those who answered “sometimes” “seldom” or “never” were regarded as “no”; and those who were unable to answer the question were excluded as missing cases. Note that the feeling of uselessness is a symptom of negative emotion in the Chinese cultural context. Anxiety and loneliness are also typical indicators of negative emotion. The correlation coefficients among these three indicators are rather high: 0.31 between anxiety and loneliness, 0.21 between uselessness and loneliness, and 0.18 between uselessness and anxiety; all of them are highly significant (p < 0.0001). The Cronbach’s alpha of these three indicators is 0.452, which is relatively small due to small number of indicators. Given that these three items have close inter-correlations and they are all symptoms or indicators of negative emotion in the Chinese cultural context, we use a variable named “negative emotion” to summarize the information collected from these three questions. Participants were coded as 1 if they answered “yes” to at least one or more of the three questions and 0 if they answered “yes” to none of the three questions. Such a measure of negative emotion was successfully employed, validated, and tested in our previous publication [44].

*Regular exercise* is assessed by the following question: “Do you exercise regularly at the present time?” Response options are “yes” and “no,” coded 1 and 0, respectively. In the CLHLS questionnaire and interviewers’ manual, regular exercise is defined as “activities for the purpose of improving health such as walking, playing ball, jogging, Tai Ji, Qi Gong, etc., excluding housework.”

*Social-leisure activities* score is based on frequency of participation in seven activities: personal outdoor activities, gardening, raising domestic poultry/pets, playing cards or mah-jongg, participating in organized social activities, reading newspaper/books, and watching TV and/or listening to the radio. Respondents who report engaging in the activity once or more per week are coded 1; otherwise, coded as 0. We then sum the seven scores (range from 0 to 7) and dichotomize the social-leisure activities score as ≥2 vs. <2 ^g^.

Note that the demographic and socioeconomic variables included as covariates in our regression models and discussed above are all associated with the dependent variables of health outcome being investigated, based on the literature [45], our understanding of the Chinese social context, and the previous publications using the CLHLS datasets [29,44]. Thus, inclusion of these variables in the regression may help to reduce or eliminate the potential confounding bias. The sample distributions of the variables included in our regression models are presented in Table 1.

**Table 1:** Sample distributions of the variables in this study, CLHLS 1998 baseline survey

**Genotypes (dominant model, see endnote b)**	**Proportion**	**Other independent variables than genotypes**	**Proportion (or mean)**
Carriers of *rs1042718* minor allele	0.56	Urban residence	0.31
Carriers of *rs*1042719 minor allele	0.72	1+ years of schooling	0.22
**Health indicators**		Currently married	0.06
MMSE score 24+	0.35	High proximity to children	0.77
MMSE score 21-23	0.14	Currently doing regular exercise	0.21
MMSE score <21	0.51	Mean of social/leisure activities score (range: 0 to 7; 27.7% ≥2; 72.3% <2)	1.01
Self-reported poor health	0.45	Negative emotion	0.54

Note: The minimum/maximum age, mean age, and standard deviation for female subjects are 90/113, 98.6, and 3.34, respectively; the corresponding figures for male subjects are 90/113, 101.0 and 2.78.

### Statistical models

We conducted logistic regressions (ordered or binary, according to the measurement of the dependent variable) to estimate the associations between carrying the *ADRB2* minor alleles and the health outcome measured by cognitive function (MMSE) and self-reported health. Wald statistical tests confirmed that the proportional odds ratio/parallel lines hypothesis is met in our ordered logistic models.

Note that the estimates of the interactions between a genotype (e.g., carrying or not-carrying the *ADRB2* minor allele in this study) and a social/behavioral factor (abbreviated as GxE hereafter) that are significantly associated with a health outcome represent a *synergistic* association. A significant *synergistic* association may not exactly reflect the true association between GxE interaction and the health outcome because the estimates may be confounded by gene and social/behavioral correlations (abbreviated as rGE hereafter). The rGE refers to the possibility that certain genotypes are correlated to social/behavioural risk factors and influence health outcomes indirectly through these pathways [46]. To estimate the association of GxE with health outcome, adjusted for the potential correlation of rGE, we further develop and empirically test in this article an innovative three-step procedure which combines standard regression and structural equations methods, based on ideas raised (not tested) in our previous publication [47]:

Step 1: *Estimate the synergistic association.* One uses standard regression methods (e.g. logistic regressions) to estimate gene-social/behavioural interaction terms, such as interaction terms between carrying or not-carrying the *ADRB2* minor allele and regular exercise or social-leisure activities or negative emotion in this study.

Step 2: *Detect rGE.* For all significant gene-social/behaviour interaction terms (i.e., synergistic association) discovered in Step 1, one uses Chi-squared test (for discrete social/behavioural factors) or the ANOVA model (for continuous social/behavioural factors) or the multiple regression model adjusted for relevant covariates to detect whether the rGE exists. In other words, one tests whether the differences in exposure to the social/behavioural factors (e.g. regular exercise or social-leisure activities or negative emotion in this study) between the carriers and non-carriers of the targeted genotype (rGE) are statistically significant.

**Figure 1:**
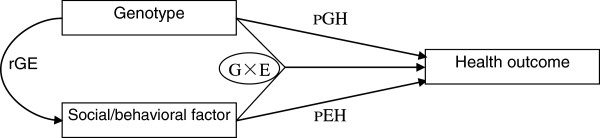
Analytical framework to explore the genotype’s direct (pGH), indirect (pEH, through its correlation with social/behavioral factors (rGE), and interactive (GxE) associations with heath outcome.

Step 3: *Path analysis for a deeper understanding.* For all significant rGE detected in Step 2, one conducts path analysis employing structural equation models, adjusted for various confounders, to further explore the direct, indirect, and interactive associations of the genetic and social/behavioural factors with the health outcome (see Figure 1 for the analytical framework).

The logistic regression analyses were performed using STATA/SE 12.0; the structural equations analyses were performed using M-Plus 6.1 [48].

## Results

### Logistic regression analysis on the associations between carrying the *ADRB2* minor alleles and the health outcomes (direct effects)

Our logistic regression analysis shows that carrying the *rs1042718* or *rs1042719* minor allele is not significantly associated with cognitive function and self-reported health at the conventional level (see models I and II in Table 2). When the (ADRB2 genotype x negative emotion) interaction term is included in the regression models, carrying the *rs1042718* minor allele is associated with cognitive function and self-reported health marginally at the p < 0.10 level (models I-A4 and II-A4 in Table 2), and carrying the *rs1042719* minor allele is associated with cognitive function marginally at the p < 0.10 level (model I-B4 in Table 2).

**Table 2:** **Odds ratios for associations with *MMSE score and Self-reported health *based on logistic regression analyses**

**Dependent variable**	**MMSE score (Good/moderate/Poor)**								**Self-reported good health**							
	**(Model I, based on ordered logistic regression)**								**(Model II based on binary logistic regression)**							
Genotype included in model	*rs1042718*				*rs1042719*				*rs1042718*				*rs1042719*			
Model code	I-A1	I-A2	I-A3	I-A4	I-B1	I-B2	I-B3	I-B4	II-A1	II-A2	II-A3	II-A4	II-B1	II-B2	II-B3	II-B4
*rs1042718* carrier (non-carrier)	1.00	0.83	0.99	1.54*					1.12	0.91	1.03	1.57*				
*rs1042719* carrier (non-carrier)					1.04	0.96	1.04	1.65*					0.97	0.73	0.77	1.14
Male (female)	1.60**	1.61**	1.59**	1.56**	1.68***	1.68***	1.68***	1.65**	1.42*	1.42	1.41	1.40	1.39	1.39	1.36	1.37
Urban (rural)	1.03	1.04	1.03	1.04	1.06	1.06	1.06	1.05	1.24	1.26	1.25	1.26	1.27	1.28	1.27	1.27
Age	0.97	0.97	0.97	0.96	0.97	0.97	0.97	0.97	1.03	1.03	1.03	1.03	1.03	1.04	1.03	1.03
≥ 1 year schooling (no)	1.91***	1.85***	1.91***	1.98***	1.85***	1.82***	1.85***	1.92***	0.85	0.82	0.85	0.87	0.90	0.86	0.93	0.91
Married (not married)	1.52	1.55	1.53	1.51	1.51	1.53	1.51	1.47	1.14	1.17	1.16	1.13	1.13	1.19	1.21	1.13
Close proximity to children (no)	0.89	0.88	0.89	0.86	0.87	0.87	0.87	0.84	0.93	0.93	0.93	0.91	0.92	0.92	0.92	0.91
Soc/leisure activity score ≥2 (<2)	1.94***	1.92***	1.89**	1.99***	1.98***	1.98***	1.97**	1.97***	2.18***	2.16***	1.86**	2.21***	2.13***	2.14***	1.19	2.12***
Regular exercise (no)	1.87***	1.21	1.87***	1.91***	1.86***	1.44	1.86***	1.90***	1.77***	1.05	1.76***	1.79***	1.79***	0.71	1.77***	1.80***
Negative emotion (no)	0.47***	0.46***	0.47***	0.73	0.48***	0.48***	0.48***	0.85	0.63***	0.62***	0.63***	0.87	0.61***	0.60***	0.62***	0.74
**GxE interaction terms**																
*ADRB2* x (exercise)		2.26**				1.44				2.68**				3.77***		
*p-value*		0.026				0.358				0.013				0.002		
*ADRB2* x (social & leisure activity)			1.06				1.01				1.36				2.29**	
*p-value*			0.873				0.978				0.398				0.034	
*ADRB2* x (negative emotion)				0.47**				0.45**				0.56*				0.77
*p-value*				0.014				0.022				0.076				0.474
LR chi2		**4.96**	0.03	**6.02**		0.84	0.00	**5.23**		6.20	0.72	3.15		9.61	4.47	5.94
Prob > chi2 (P)		**0.03**	0.87	**0.01**		0.36	0.98	**0.02**		**0.01**	0.40	0.08		**0.00**	**0.03**	**0.48**
−2LL (-2 log Likelihood)	1308.6	1303.6	1308.6	1302.6	1299.9	1299.1	1299.9	1294.7	907.5	901.3	906.8	904.4	901.1	891.5	896.6	900.6

Notes: (1) *p < 0.1, **p < 0.05, ***p < 0.01. For all interaction terms tested, we also listed the exact p values. (2) LR chi2 is computed using model -A1 or –B1 as the benchmark.

### Logistic regression analysis on association between the GxE interactions and health outcomes (interactive effects)

Following the standard Aiken and West procedure [49], we conducted blocked multiple regressions and Chi-square tests to examine whether the difference in likelihood ratios between the full models including the interaction block (models -A2, -A3, -A4 and -B2, -B3, -B4 in Table 2) and the models without the interaction block (models -A1 and -B1 in Table 2) are statistically significant. Such tests also inform whether the block of interactions included in the regression is statistically significant. The results of these additional tests are listed in the last three rows of Table 2 and are consistent with the estimates of the statistical significance level of the interaction terms of the regressions. The significant results of these additional tests also imply that the likelihood of a type I error in our estimates of the interaction terms may be small [50].

The GxE interaction between carrying the *rs1042718* minor allele and regular exercise is significantly and positively associated with cognitive function (*p* <0.05; model I-A2, Table 2) and self-reported good health (*p* <0.05; model II-A2, Table 2). The GxE interaction between carrying the *rs1042719* minor allele and regular exercise is also significantly and positively associated with self-reported good health (*p* <0.01; model II-B2, Table 2). Furthermore, GxE interaction between carrying the *rs1042719* minor allele and higher social/leisure activities score is significantly and positively associated with the likelihood of self-reported good health (*p* <0.05; model II-B3, Table 2). We discovered that the estimates of the GxE interaction terms between carrying the rs1042718 or rs1042719 minor allele and negative emotion were statistically significant (OR = 0.47 with p <0.05 or OR = 0.45 with p <0.05).

The Chi-squared tests ruled out rGE correlation between carrying the *ADRB2* minor allele and regular exercise or social/leisure activities (Tables 3 and 4). Thus, the estimates of the GxE interaction terms between carrying the ADRB2 minor alleles and regular exercise or social/leisure activities (ref. models I-A2, II-A2, II-B2 and II-B3 in Table 2) discussed above represent the true associations between the GxE interactions and the health outcome.

**Table 3:** **Chi-square tests to assess the correlations between carrying *rs1042718 *minor allele and behavioral factors**

**Social/behavioral factor**	**Regular exercise**		**Negative emotion**	
	**Yes**	**No**	**Yes**	**No**
% carriers of *rs1042718* allele	56.8%	55.3%	52.2%	59.3%
*p* of Chi-square test	*p* = 0.723		*p* = 0.055	
Conclusion concerning rGE	rGE does not exist		rGE exists marginally	

**Table 4:** **Chi-square tests to assess the correlations between carrying *rs1042719 *minor allele and behavioral factors**

**Social/behavioral factor**	**Regular exercise**		**Soc/leisure activity**		**Negative emotion**	
	**Yes**	**No**	**Score** ≥ **2**	**Score < 2**	**Yes**	**No**
% carriers of *rs1042719* allele	71.4%	71.9%	70.3%	72.5%	69.0%	74.6%
*p* of Chi-square test	*p* = 0.886		*p* = 0.513		*p* =0.094	
Conclusion concerning rGE	rGE does not exist		rGE does not exist		rGE exists marginally	

### Path analysis employing the structural equations method

As shown in Tables 3 and 4, the Chi-squared tests show that the rGE correlation between carrying ADRB2 minor alleles and negative emotion cannot be ruled out (the estimates are marginally significant, p <0.1). Moreover, we found that carrying the rs1042718 or rs1042719 minor allele is significantly and negatively associated with negative emotion (p <0.05), controlling for the socio-demographic characteristics of age, gender, rural/urban residence, education, family/social connections (marital status, proximity to children, and social/leisure activities score) and health practice (regular exercise) (see Table 5). Thus, the statistically significant GxE interactive terms between the ADRB2 genotypes and negative emotion presented in models I-A4 and I-B4 in Table 2 may be confounded by the rGE correlation between carrying ADRB2 minor alleles and negative emotion, and the ADRB2 genotypes may have an indirect association with cognitive function through negative emotion (see Figure 1 and its associated discussions on the three-step procedure in Section 2.3).

**Table 5:** **Odds ratios of associations with *negative emotion, *based on binary logistic regression analysis**

**Dependent variable**	**Negative emotion (yes or no)**								
Indepen-dent variable	Carrier of ADRB2 allele (non-carrier)	Male (female)	Urban (rural)	Age	≥ 1 year schooling (no)	Married (not married)	Close proximity to children (no)	Soc/leisure act. score ≥2 (<2)	Regular exercise (no)
*rs1042718*	0.69**	0.70*	0.86	1.03	1.08	1.5	0.91	0.67**	0.79
*rs1042719*	0.69**	0.74	0.86	1.02	1.03	1.47	0.93	0.69**	0.78

Note: *p < 0.1, **p < 0.05, ***p < 0.01. For all interaction terms tested.

We conducted path analysis employing the structural equations method to further explore *ADRB2* genotype’s direct association with MMSE, indirect association with MMSE through its correlation (rGE) with negative emotion, and the association between *ADRB2* genotype-negative emotion interactions and MMSE (see Section 2.3 and Figure 1 for the analytical framework). The estimates of the odds ratios of the direct association of the ADRB2 genotypes with MMSE score and negative emotion based on the structural equations (see Table 6) are pretty close to the corresponding estimates of the odds ratios based on logistic regressions (see Table 2). However, the structural equations analysis provides additional new results and insights. The odds ratios of indirect association between carrying the *rs1042718* or *rs1042719* minor allele and cognitive function through rGE correlation with negative emotion is 1.12 or 1.04, respectively (see the last line of Table 6). Although the M-Plus 6.1 software did not provide an estimate of the p value of the indirect association (= a x b, see Table 6), these odds ratios are unlikely to be statistically significant because they are rather close to 1.0. The results demonstrated that, after adjusting for the confounding effects of the rGE correlation, the GxE interaction between carrying the *rs1042718* or *rs1042719* minor allele and negative emotion is significantly and negatively associated with cognitive function with an odds ratio of 0.47 (p <0.05) or 0.46 (p <0.05), respectively (see Table 6) ^h^, which are almost the same as those estimated by the logistic regression (see Table 2).

**Table 6:** Estimates of coefficients and odds ratios, based on the structural equations analysis

**Model**	**SEM model including *rs1042718 *and negative emotion**			**SEM model including *rs1042719 *and negative emotion**		
	Independent variable	Coef.	Odds ratio	Independent variable	Coef.	Odds ratio
**Depend. Variable: MMSE Score (Direct association)**	*rs1042718 carrier (no)*	**c** =0.45*	1.58*	*rs1042719 carrier (no)*	**c** =0.51**	1.67**
	Age	−0.04	0.96	Age	−0.04	0.96
	Male (female)	0.42**	1.52**	Male (female)	0.47**	1.59**
	Urban (rural)	0.06	1.06	Urban (rural)	0.07	1.07
	≥1 year of schooling (no)	0.72***	2.06***	≥1 year of schooling (no)	0.69***	2.00***
	Married (not-married)	0.40	1.49	Married (not-married)	0.38	1.46
	Social/leisure activity ≥2 (<2)	0.70***	2.01***	Social/leisure activity ≥2 (<2)	0.69***	2.00***
	Regular exercise (no)	0.66***	1.93***	Regular exercise (no)	0.65***	1.92***
	Negative emotion (no)	**b** = -0.31	0.74	Negative emotion (no)	**b** = -0.16	0.85
	**Significant GxE interaction**					
	(*rs1042718*) x (Negative emotion)	−0.75**	0.47**	(*rs1042719*)x(Negative emotion)	−0.77**	0.46**
**Depend. Variable: Negative emotion (controlling for the confounding effects)**	*rs1042718 carrier (no)*	**a** = -0.34**	0.71**	*rs1042719 carrier (no)*	**a** = -0.33*	0.72*
	Age	0.03	1.03	Age	0.02	1.02
	Male (female)	−0.33	0.72	Male (female)	−0.28	0.76
	Urban (rural)	−0.13	0.88	Urban (rural)	−0.13	0.88
	≥1 year of schooling (no)	0.08	1.08	≥1 year of schooling (no)	0.03	1.03
	Married (not-married)	0.38	1.46	Married (not-married)	0.36	1.44
	Social/leisure activity ≥2 (<2)	−0.37**	0.69**	Social/leisure activity ≥2 (<2)	−0.34*	0.72*
	Regular exercise (no)	−0.26	0.77	Regular exercise (no)	−0.26	0.77
**Indirect association of *ADRB2 with *MMSE**	Indirect association of carrying *rs1042718* with MMSE through its rGE with negative emotion	**a x b** = (-0.34) x (-0.31) = 0.11	1.12 = exp(0.11)	Indirect association of carrying *rs1042719* with MMSE through its rGE with negative emotion	**a x b** = (-0.33) x (-0.13) = 0.04	1.04 = exp(0.04)

Note: (1) Following methodology of structural equations models concerning indirect effects of the explanatory variable on the dependent variable via another related explanatory variable [51-54], the indirect effects of carrying the *ADRB2* minor allele on MMSE through its effects on negative emotion can be estimated by “a x b”. (2) *p < 0.1, **p < 0.05, ***p < 0.01.

### A more intuitive way to present and interpret the association between GxE interaction and the health outcome

An interaction between a social/behavioral factor and a genotype is present if the association between the social/behavioral factor and a health outcome indicator differs among individuals with different genotypes, or if the association between the genotype and a health outcome indicator differs among individuals with different social/behavioral factors [45]. Consequently, in addition to looking at the odds ratios of the GxE interaction terms presented in Tables 2 and 6, another more intuitive way to present and interpret the association between GxE interaction and the health outcome is to compare the relative risks of health outcome between those who are exposed or not-exposed to the social/behavioural factor within the group of carriers of the genotype and within the group of non-carriers.

Table 7 shows that, among non-carriers of the *rs1042718* minor allele, regular exercise was not significantly associated with increased odds ratios for cognitive function or self-reported good health. Among carriers of *rs1042718* minor allele, however, regular exercise substantially increased the odds ratio associated with cognitive function by 173.5% and the odds ratio associated with self-reported good health by 181.4%. Table 8 shows a similar pattern. Among non-carriers of the *rs1042719* minor allele, regular exercise and social/leisure activity were not significantly associated with odds ratios for self-reported good health. Among carriers, regular exercise substantially increased the odds ratio associated with self-reported good health by 167.7% and a higher social/leisure activities score increased the odds ratio associated with reporting good health by 172.5%. Clearly, the positive associations of regular exercise and social-leisure activities with cognition and self-reported good health are much stronger among carriers of *rs1042718* or *rs1042719* alleles, compared to non-carriers.

**Table 7:** **Differences in odds ratios (*OR***_***EG***_**) of MMSE score and self-reported health by social/behavioral exposure status(E) and *rs1042718 *minor allele carrier status(G)**

**Dependent variable**	**MMSE score**			**Self-reported good health**		
	**(Model I-A2,ref. Table **2**)**			**(Model II-A2,ref. Table **2**)**		
G: genotypic status	E: regular exercise			E: regular exercise		
	No	Yes	% difference	No	Yes	% difference
	(E = 0)	(E = 1)	E = 1 vs. 0	(E = 0)	(E = 1)	E = 1 vs. 0
*OR*_*E0*_ of Non-carrier *rs1042718* (G = 0)	1.00	1.21	21.0%	1.00	1.05	5.0%
*OR*_*E1*_ of Carrier *rs1042718* (G = 1)	0.83	2.27***	173.5%	0.91	2.56***	181.4%
GxE interaction	2.26** (1.10 ~ 4.62)			2.68** (1.23 ~ 5.85)		

Notes: (1) The estimates of *OR*_*10*_, *OR*_*01*_ and the “odds ratio of GxE interactions (*ORIT*)” are taken from models I-A2 and II-A2 of logistic regression analysis presented in Table 2. (2) *p < 0.1, **p < 0.05, ***p < 0.01. (3) Because *ORIT* = *OR*_11_/(*OR*_10_⊠*OR*_01_) [48,55], *OR*_*11*_ *= OR*_*10*_*OR0*_*01*_*ORIT.* However, we do not know the statistically significant level (i.e., p value) of the OR_*11*_ estimated based on OR_*10*_, OR_*01*_ and *ORIT* normally produced by the statistical software. Thus, we alternatively estimated OR_*10*_, OR_*01*_ and OR_*11*_ and their p values by setting up three exclusive dummy variables V10, V01 and V11 (without interaction term) in the regression equation: V10 =1 if E = 1 and G = 0; V01 = 1 if E = 0 and G = 1; and V11 = 1 if E = 1 and G = 1, while considering V00 (E = 0 and G = 0) as the reference group. In such alternative regression, the OR_*11*_, OR_*10*_, OR_*01*_ and their significant levels are estimated and the *ORIT* can be calculated based on estimates of the OR_*10*_, OR_*01*_ and OR_*11*_. Note that the OR_*10*_, OR_*01*_, OR_*11*_ and *ORIT* estimated in such an alternative regression are exactly the same as those estimated in the normal procedure (with two dummy variables and one interaction term) adopted by the statistical software.

**Table 8:** **Differences in odds ratios (*OR***_***EG***_**) of self-reported good health by regular exercise and social/behavioral exposure status(E) and *rs1042719 *minor allele carrier status(G)**

**Dependent variable**	**Self-reported good health**			**Self-reported good health**		
	**(Model II-B2,ref. Table **2**)**			**(Model II-B3,ref. Table **2**)**		
G: genotypic status	E: regular exercise			E: social & leisure activity		
	No	Yes	% difference	No	Yes	% difference
	(E = 0)	(E = 1)	E = 1 vs. 0	(E = 0)	(E = 1)	E = 1 vs. 0
*OR*_*E0*_*of Non-carrier rs1042719* (G = 0)	1.00	0.71	−29.0%	1.00	1.19	19.0%
*OR*_*E1*_*of Carrier rs1042719* (G = 1)	0.73	1.95**	167.7%	0.77	2.10***	172.5%
GxE interaction	3.77*** (1.63 ~ 8.72)			2.29**(1.07 ~ 4.92)		

Notes: (1) The estimates of *OR*_*10*_, *OR*_*01*_ and “GxE interactions” are taken from models II-B2 and II-B3 of logistic regression analysis presented in Table 2. (2) *p < 0.1, **p < 0.05, ***p < 0.01. (3) The same as note (3) in Table 7.

As shown in Table 9, among the non-carriers of *rs1042718* or *rs1042719* minor allele, negative emotion decreased the odds ratio associated with cognitive function by -26.0% or -15% and the estimates are not statistically significant. However, among the carriers of *rs1042718* or *rs1042718* minor allele, negative emotion much more substantially decreased the odds ratio associated with cognitive function by -65.7% or -61.1% and the estimates are statistically significant.

**Table 9:** **Differences in odds ratios (*OR***_***EG***_**) of MMSE score by negative emotion(E) and the minor allele carrier status(G)**

***rs1042718***				***rs1042719***			
**G: genotypic status**	**E: negative emotion**			**G: genotypic status**	**E: negative emotion**		
	**No**	**Yes**	**% difference**		**No**	**Yes**	**% difference**
	**(E = 0)**	**(E = 1)**	**E = 1 vs. 0**		**(E = 0)**	**(E = 1)**	**E = 1 vs. 0**
*OR*_*EG*_*of non-carrier rs1042718* (G = 0)	1.00	0.73	−26.0%	*OR*_*EG*_*of non-carrier rs1042719* (G = 0)	1.00	0.85	−15.0%
*OR*_*EG*_*of carrier rs1042718* (G = 1)	1.54*	0.53***	−65.7%	*OR*_*EG*_*of carrier rs1042719* (G = 1)	1.65*	0.63*	−61.1%
GxE interaction	0.47** (0.25 ~ 0.86)			GxE interaction	0.45** (0.23-0.89)		

Notes: (1) The estimates of OR10, OR01 and “GxE interactions” are taken from models I-A4 and I-B4 of logistic regression analysis presented in Table 2. (2) *p < 0.1, **p < 0.05, ***p < 0.01. (3) The same as note (3) in Table 7.

## Discussion

The findings reported in this article are generally consistent with the other studies which demonstrated that *ADRB2* genotypes were associated with resistance to stresses [5], psychological disorders [9-13], and depression [56,57]. Our present study has made unique and useful contributions to the literature in three aspects. First, this is the first study on the associations of carrying the *ADRB2* minor alleles and the GxE interactions with health outcomes at advanced ages based on a relatively large sample of 877 oldest-old participants aged 90+ ^i^. Previous relevant studies on humans in this sub-field focused on younger adults (aged 20 to 70) and had very small sample sizes from 22 to 210 individuals (as reviewed in the introduction section). Second, for the first time, our findings demonstrated that some social/behavioral factors have much stronger associations with cognitive function and self-rated health at advanced ages among *ADRB2* minor allele carriers compared to non-carriers. This could imply that personalized preventive medicine based on individuals’ genetic profiles, while strictly protecting privacy, may be helpful in increasing cost-effectiveness and efficiency to promote healthy aging [58]. Third, in addition to estimating the main/direct associations, we also estimated the indirect associations of carrying the *ADRB2* minor allele with cognitive function, through its rGE correlation with negative emotion. Adjusted for the confounding effects of the rGE correlation, we estimated the association between the GxE interaction [(carrying the *ADRB2* minor allele) x (negative emotion)] and cognitive function. To our knowledge, this is the first such attempt based on our innovative three-step procedure which combines the logistic regression and structural equations methods (see Table 6) to explore the impacts of *ADRB2* genotypes on cognitive function.

Our study also has important limitations and unanswered questions. We have only limited measures of self-rated health outcome collected in the CLHLS, which is a typical demographic and epidemiological survey focusing on determinants of healthy longevity rather than a special medical study. Furthermore, our exploratory association study did not investigate the causal effects of biological mechanisms underlying the new findings. Future investigations may answer why the positive associations of regular exercise and social & leisure activities with cognition and self-rated health, and the negative association of negative emotion on cognition, are much stronger among carriers of the *ADRB2* allele than among non-carriers. Because the combined genotype and phenotype data were available for 877 oldest-old aged 90+ only, we restricted our present study within the advanced age range and we are not able to conduct replication and validation studies.

The biostatistics literature states that measuring multiple health outcomes and explanatory factors can lead to multiple comparisons and p values may need to be adjusted by the Bonferroni method or other similar method; however, if the null hypotheses are not independent and the multiple comparisons are complementary, then the p values may not need to be corrected [59,60]. Our multiple logistic regression and structural equations models estimates with a statistical significance level of 1% (*p* < 0.01) or 5% (*p* < 0.05) may not need to be corrected, because the two outcome indicators and multiple explanatory factors in our case are complementary and not independent of each other. For example, both SNPs of *rs1042718* and *rs1042719* are highly correlated and in strong linkage disequilibrium (70%) [8]. Accordingly, there is no need for correction of tests of association with different SNPs. The two health indicators (cognitive function and self-reported health) are highly correlated, the social/behavioral factors (regular exercise, social & leisure activity and negative emotion) are highly correlated. Furthermore, in our exploratory study, in which data are collected with an objective but not with a pre-specified and confirmatory hypothesis, multiple test adjustments are not strictly required [61]. Obviously, we do not need to correct the p values in this study, because the multiple tests involved are not independent each other and our analyses are explanatory. We must be, however, cautious in interpreting the estimates of our present study and treat them as exploratory findings rather than any final conclusive results because they could still involve some false positive errors due to the multiple comparisons, sample size, and lack of replication studies.

## Conclusions

Our logistic regression analysis discovered that, adjusted for various potentially confounding factors of demographics, socioeconomic status, family and social connections/support, and health practice behaviors, the GxE interaction between regular exercise and carrying the *rs1042718* minor allele is significantly associated with cognitive function score (*p* <0.05); GxE interactions between regular exercise and carrying *rs1042718* or *rs1042719* minor allele are significantly associated with self-reported good health (*p* <0.05 or *p* <0.01); and GxE interactions between social-/leisure activities and carrying *rs1042719* minor allele are significantly associated with self-reported good health (*p* <0.05) (see Table 2).

Our structural equations path analysis shows that GxE interactions between negative emotion and carrying the *rs1042718* or *rs1042719* minor allele are significantly associated with cognitive function (p <0.05 or p <0.05). We also found that carrying the *rs1042718* minor allele or *rs1042719* minor allele is significantly associated with negative emotion (*p* <0.05). The rGE correlation between carrying the *rs1042718* or *rs1042719* minor allele and negative emotion is indirectly associated with cognitive function, but the estimates are not statistically significant (see Table 6).

We also discovered that the associations between social/behavioral factors (including regular exercise, social/leisure activities and negative emotion) and cognitive function or self-reported health are much stronger among carriers of the *ADRB2* minor alleles than among the non-carriers (see Tables 7, 8, and 9). These findings imply that health promotion programs considering individuals’ genetic profiles (with appropriate protection of privacy/confidentiality) would yield increased benefits and reduced costs to the programs and their participants.

As detailed in the discussion section, while we are satisfied with the interesting and unique findings reported in this article, our study also has important limitations and unanswered questions; our findings are typically exploratory rather than any kind of causal conclusions. Clearly, additional in-depth epigenetics studies with more sophisticated psychological indicators are needed to develop a deeper understanding of the biological mechanisms and causalities of how and why carrying the *ADRB2* minor alleles and their interactions with social/behavioral factors may affect health outcome at old ages. Further studies need to extend the analysis to explore the effects of GxG interactions between ADRB2 genotypes and the other genotypes associated with health and longevity at old ages (e.g. APOE4 and FOXO genotypes), and to cover all elderly age groups (i.e., from age 65 to 110) to fully understand the process of healthy aging in a life course perspective. We also hope that other studies on healthy longevity involving similar or substantially larger samples of the oldest-old aged 90+ will replicate and validate our exploratory findings.

### Endnotes

^a^These 22 provinces are: Liaoning, Jilin, Heilongjiang, Hebei, Beijing, Tianjing, Shanxi, Shaanxi, Shanghai, Jiangsu, Zhejiang, Anhui, Fujian, Jiangxi, Shangdong, Henan, Hubei, Hunan, Guangdong, Guangxi, Sichuan, Chongqing.

^b^In a dominant model, any genotype that contains 1 or 2 copies of the minor allele is coded as 1; and otherwise the genotype that does not contain copy of the minor allele is coded as 0 (i.e. mm, Mm = 1, MM = 0. Here, M: major allele, m: minor allele.). For the recessive model, the homozygous mm genotype is coded as 1 and 0 otherwise (i.e. mm = 1, Mm, MM = 0). In an additive model, genotype MM is coded as 0, Mm, 1, mm, 2.

^c^Note that Zhao et al. [8] basically used genotype data from 893 long-lived Han Chinese CLHLS participants’ blood dry-spot samples collected in 1998 at baseline of CLHLS. At the final stage of revisions and resubmission of the article, Zhao et al. added 70 long-lived individuals’ full blood samples collected from the normal health examinations in the hospitals (rather than CLHLS), mostly for some biological functional analysis to strengthen the explanations of the results. Thus, the final total number of long-lived samples was 963, as stated in Zhao et al. [8]. However, the lately-added 70 long-lived cases were NOT CLHLS participants and the phenotype data needed for the GxE analysis are not available from them, and thus we did not include them in this paper. Furthermore, there are 16 long-lived CLHLS participants whose genotype data are available but some of their main phenotypic variables are missing, and thus, we used 877 valid cases of long-lived CLHLS participants in this study.

^d^MMSE is used internationally in healthy aging investigations and works well in studies that are not specialized psychological research.

^e^We also tried the continuous MMSE scores, the four categories (<10, 10-17, 18-23, > = 24) of MMSE scores and the dichotomized MMSE scores. The results of the continuous and the alternative classifications of MMSE scores are generally consistent with the estimates using the three categories of the MMSE scores, while the significance level reduced moderately in the continuous model and slightly in the four categories and dichotomized models, as compared to the three-category model we adopted.

^f^About 78% of the Chinese oldest-old aged 90+ had no schooling.

^g^Participation in the social-leisure activities depends on senior adults’ interests and it is also subject to time limit. Thus, it is not necessarily the case that the larger number of social-leisure activities an elder participated in, the more active he/she is, and it may not make sense to use a continuous variable to measure the social-leisure activities. After trying different combinations, we found that dichotomization of the social-leisure activities score as ≥2 vs. <2 is the best choice.

^h^Note that in the structural equation model (SEM) and M-Plus software used for this study, one needs to create a new variable for the interaction term between the genetic variant and social/behavioral factor [51]. We use the maximum likelihood estimation with the Monte-Carlo option to estimate the coefficient of the interaction term variable.

^i^We conducted the statistical analysis models by pooling males and females together, while controlling for gender as a covariate. We also tried the logistic regression analysis for males and females separately and the results are basically consistent with our presented two-genders combined models; the statistical significance levels were reduced substantially due to sample size reduction.

## Competing interests

The authors declare that they have no competing interests.

## Authors’ contributions

YZ designed the research; supervised data collection and data analysis, and wrote the paper; XLT designed the genetic research and supervised the lab work; LGC, QSF, HSC and KS analyzed data; LJX, LGC, FYZ, HSC, SK performed data collection; LZ and HQC performed lab tests; ZY, JG and WT contributed to sample storage; QHT, SGG, ZY, JG, WT, XLT and ERH suggested revisions of the paper. All authors read and approved the final manuscript.

## Pre-publication history

The pre-publication history for this paper can be accessed here:

http://www.biomedcentral.com/1471-2318/13/91/prepub
